# Transcutaneous Closure of Persistent Ductus Arteriosus: Complication Rates and Long-term Follow-up, a Single-Centre Retrospective Study

**DOI:** 10.1016/j.cjcpc.2025.04.001

**Published:** 2025-04-11

**Authors:** Ian Scott Kendall, Gail Davison, Neil Kennedy, Brian McCrossan

**Affiliations:** a Paediatric Cardiology Department, Royal Belfast Hospital for Sick Children, Belfast, United Kingdom; b Wellcome Wolfson Institute of Experimental Medicine, Queens University Belfast, Belfast, United Kingdom

## Abstract

**Background:**

Transcutaneous closure of patent ductus arteriosus (PDA) in childhood is a common procedure. Long-term follow-up by paediatric cardiologists is variable. Identification and classification of postoperative complications may enable targeted follow-up and timelier discharges. This study aimed to characterize complication rates and assess discharge timing.

**Methods:**

This is a single-centre retrospective study of paediatric patients (aged 0-15 years) who underwent a transcutaneous closure of a PDA between January 2006 and December 2015.

**Results:**

A total of 156 patients who underwent interventional occlusion of a PDA were included. Complications were seen in 18 of 156 (12%) patients. High-grade complications occurred in 8 of 156 (5.1%) patients; these included device embolization, failure requiring surgical closure, or repeated interventional closure. Moderate to low-grade complications including flow acceleration in the aorta and left pulmonary artery (LPA) occurred in 10 of 156 (6.4%) patients. Fourteen of 18 (77%) complications were immediately apparent. Late mild to moderate obstruction of the descending aorta or LPA occurred in 3 of 156 (2%) patients. Later obstruction occurred in the Amplatzer ductal occluder 1 (ADO1) group only with large (4.5-5 mm) ducts. The average follow-up time for all patients was 81 (±47) months. Younger age at insertion and larger size of ADO1 devices were associated with later obstruction.

**Conclusions:**

In our cohort, PDA occlusion was associated with a 5.1% major complication rate, which is evident within 24 hours; a further 2% (all treated with ADO1 devices) developed between mild and moderate aortic or LPA obstruction at least 1 year after the procedure. To date, this has not required intervention. It may therefore be prudent to continue longer-term surveillance of patients who have undergone PDA occlusion with the ADO1 device.

## Résumé

**Contexte:**

La fermeture percutanée du canal artériel persistant (CAP) pendant l’enfance est une intervention courante. Le suivi à long terme varie d’un cardiologue pédiatrique à l’autre. Le dépistage et la classification des complications postopératoires pourraient permettre de cibler le suivi et d’accélérer l’obtention du congé de l’hôpital. Cette étude visait à caractériser les taux de complications et à évaluer le moment du congé de l’hôpital.

**Méthodologie:**

Il s’agit d’une étude rétrospective menée dans un seul centre chez des enfants (âgés de 0 à 15 ans) ayant subi une fermeture percutanée du CAP entre janvier 2006 et décembre 2015.

**Résultats:**

Au total, 156 patients ayant subi l’intervention ont été inclus. Des complications ont été observées chez 18 patients sur 156 (12 %). Il s’agissait de complications de haut grade chez 8 patients sur 156 (5,1 %), et comprenaient l’embolisation du dispositif, l’échec de l’intervention nécessitant une fermeture chirurgicale ou la répétition de l’intervention. Les complications de grade faible à modéré observées comprenaient l’augmentation du débit dans l’aorte et l’artère pulmonaire gauche (APG), et ont été observées chez 10 patients sur 156 (6,4 %). Quatorze complications sur 18 (77 %) ont été visibles immédiatement. Une obstruction légère à modérée et tardive de l’aorte descendante ou de l’APG a été observée chez 3 patients sur 156 (2 %). Une obstruction tardive est survenue dans le groupe traité à l’aide du dispositif ADO1 (*Amplatzer Ductal Occluder 1*) et uniquement avec les dispositifs de grand diamètre (4,5 à 5 mm). La durée moyenne du suivi pour tous les patients a été de 81 (± 47) mois. Un âge plus précoce au moment de l’insertion et les dispositifs ADO1 de plus grand diamètre ont été associés à une obstruction tardive.

**Conclusions:**

Dans notre cohorte, la fermeture du CAP a été associée à un taux de complications majeures visibles dans les 24 heures de 5,1 %. D’autres complications, notamment des obstructions légères à modérées de l’aorte ou de l’APG ont été observées au moins 1 an après l’intervention dans 2 % des cas. À ce jour, aucune intervention n’a été nécessaire. Il est donc prudent de poursuivre à plus long terme la surveillance des patients qui se prêtent à une fermeture du CAP à l’aide du dispositif ADO1.

Transcutaneous device closure of patent ductus arteriosus (PDA) has become the standard of care.^1^ Its use has expanded to smaller patients with it being regularly used in patients as small as 700 g in some centres.^1^

Possible adverse events include embolization of the device, occlusion failure, haemolysis, aortic obstruction, left pulmonary artery (LPA) turbulence, and vascular injury.^2^^,^^3^ These complications are screened for during the procedure via fluoroscopy and immediately after the procedure with echocardiography. After discharge, patients are followed up in the outpatient clinic. Current guidelines would suggest regular echocardiographic review in the first 2 years and then every 5 years lifelong.^4^ Conversely, given that significant complications tend to be identified either during the procedure itself or in the days to weeks thereafter, there has been a call by some to consider earlier discharge. Some recommend discharge as early as 3 months after the procedure assuming stable echocardiographic findings.^5^^,^^6^

This study aimed to characterize complication rates of this procedure, identify timing of complications, and provide recommendations to safely discharge patients from ongoing cardiology follow-up.

## Methods

Patients (aged 0-15 years) who underwent a transcutaneous closure of a PDA between January 2006 and December 2015 in our centre were included. Data were gathered from the departmental database (Heartsuite; Systeria, Glasgow, UK). Patients who had significant additional congenital heart disease or those aged 16 years or more were excluded. Complications were classified according to severity: low (mild), medium (possible clinical significance but no reintervention), or high severity (reintervention required). Complications were also classified according to timing: early (within 24 hours), midterm (≤2 years), or long term (2 years). These periods were chosen to highlight complications that may have been missed had the patient been discharged from cardiology follow-up at or before 2 years.

Data collected include age, weight, sex, type of closure device/coil, Krichenko grading of duct, postprocedural echo, follow-up echo findings, and length of follow-up. Follow-up intervals varied but included multiple visits in the first year after ductal closure and were less frequent thereafter. The discharge was decided on by the attending consultant.

Follow-up data up to November 2022 were used in the analysis. Descriptive statistics were used in the data analysis. In patients with Amplatzer ductal occluder 1 (ADO1) devices (St. Jude Medical, St. Paul, MN), risk factors were analysed to establish an association between these factors and late aortic/LPA turbulence (defined as a maximum velocity [VMAX] ≥2.0 m/s identified 1 year or later after insertion). The χ^2^ test and the Fisher exact test were used for categorical variables, and the independent samples Student *t* test was used for continuous variables. All tests were 2-sided; statistical analysis was performed on SPSS version 29 (IBM, New York, NY).

## Results

### Patients and procedures

A total of 188 patients aged 0-15 years were identified who had undergone transcutaneous PDA closure. Patients who had additional significant congenital heart disease requiring follow-up (23) and patients lost to follow-up (9) were excluded; therefore, 156 patients were included in the analysis.

The mean age and weight at the time of procedure were 43.8 months (standard deviation [SD] ±33 months) and 15.8 kg (SD ±8.5 kg, range = 3.9-80 kg), respectively. A total of 106 (68%) patients were female and 50 were male. Devices used were Cook Coils (Cook Medical Inc, Bloomington, IN) (CC) = 86/156 (56%) and ADOs (St. Jude Medical): ADO1 = 64/156 (41%) and ADO2 = 4/156 (2.5%), or a combination of ADO and CC = 2/156 (1.2%). Thirteen 3-mm, sixty-four 5-mm, six 6.5-mm, and 4 multiple combinations of CC were inserted. Seven 6/4 mm, eight 10/8 mm, and forty-nine 8/6 mm ADO1 devices were inserted. Two 4/6 mm and two 5/6 mm ADO2 devices were inserted. The mean follow-up period was 81 months (SD ±47 months). The mean number of follow-up attendances was 5 (SD ±2) (see Table 1).

**Table 1:** Patient and device characteristics

Patient characteristics	All patients, n (% or range)					Size range (mm)
Female sex	106 (68)					
Mean age at procedure (mo)	44 (5-190)					
Mean weight at procedure (kg)	15.8 (3.9-80)					
Cook Coil	86 (56)					3 × 5 to 8 × 6
ADO1	64 (40)					6/4 to 10/8
ADO2	4 (3)					4/6 to 5/6
Combination	2 (2)					
Mean follow-up time (mo)	81 (9-176)					
Discharged at the time of data collection	118 (75)					
Krichenko (n)	A 79	B 3	C 11	D 2	E 57	

ADO1, Amplatzer ductal occluder 1; ADO2, Amplatzer ductal occluder 2.

### Complications

#### Grading

Complications were seen in 18 of 156 (12%) patients. No procedural deaths or significant vascular trauma was recorded. Complications were classified as low, medium, and high grade. High-grade complications included embolization of the device/coil, failure to close the PDA requiring surgery, or residual shunt requiring further transcutaneous occlusion at a subsequent cardiac catheterization. High-grade complications occurred in 8 of 156 (5.1%) patients; these included device embolization (3 of 156, 1.9%), failure to close PDA requiring surgical ligation (2 of 156, 1.3%), and residual shunt requiring repeat transcutaneous procedure (3 of 156, 1.9%). No patients developed significant haemolysis.

Moderate and low-grade complications did not require further intervention but ongoing surveillance. One patient had evidence of aortic arch obstruction with a VMAX = 3.1 m/s, which was graded as a moderate complication. Four of 156 (2.5%) patients developed mild LPA turbulence (VMAX 2.0-2.7 m/s), and 5 of 156 (3.2%) patients developed mild descending aortic obstruction (VMAX 2-2.9 m/s).

#### Timing

Timing of the complications was also assessed. Complications were graded as early (apparent in the first 24 hours), midterm (after 24 hours and before 2 years), or long term (greater than 2 years to become apparent). Immediate complications were evident in 15 of 156 (10%) patients; these included all the high-grade complications as well as the moderate complication.

Of the 4 patients who developed LPA turbulence, 3 developed it in the short term and 1 in the long term. The patient who developed LPA turbulence in the long term (VMAX 2.3 m/s) also developed aortic turbulence (VMAX 2.9 m/s). Of the 6 patients who developed aortic turbulence, 3 developed it in the short term, 1 in the midterm, and 2 in the long term.

Medium- and long-term aortic obstruction was seen in the ADO1 group only (see Table 2).

**Table 2:** Complications classification by severity and timing

Patient	Age at closure (mo)	Weight at closure (kg)	Device (size mm)	Krichenko grade	Duct size (mm)	Complication	Timing of complication identified after procedure (S/M/L)	Severity
1	6	5.3	CC (8)	E	2.5	Moderate residual shunt (further 3-mm CC implanted)	24 h (S)	High
2	16	11.1	ADO1 (8/6)	A	2.9	Mild aortic turbulence (VMAX 2 m/s)	24 h (S)	Low
3	38	11.4	CC (6.5)	A	2	Mild LPA turbulence (VMAX 2 m/s)	24 h (S)	Low
4	30	14.3	CC (5/5)	A	2.3	Embolization (replaced with ADO1 8/6)	During procedure (S)	High
5	52	17.5	CC (3)	D	1.2	Embolization (replaced with CC 5/5)	During procedure (S)	High
6	8	7.08	ADO1 (10/8)	C	4.2	Ligation	During procedure (S)	High
7	13	10.4	ADO1 (10/8)	A	4.5	Mild aortic turbulence (VMAX 2.5 m/s)	3 y (L)	Low
8	17	10.2	CC (8)	A	3.0	Moderate residual shunt (further 3-mm CC implanted)	During procedure (S)	High
9	22	10.8	ADO1 (10/8)	C	5.1	Mild aortic turbulence (VMAX 2.9 m/s) and mild LPA turbulence (VMAX 2.3 m/s)	6 y (L)	Low
10	74	21.3	CC (3)	E	1.4	Embolization replaced with (6/4)	During procedure (S)	High
11	82	22.3	CC (5)	E	2.1	Moderate residual shunt, required further 6 × 4 ADO1	During procedure (S)	High
12	42	13.6	CC (8)	E	2.8	Ligation	During procedure (S)	High
13	13	9.7	ADO1 (6/4)	A	2.8	Mild LPA turbulence (VMAX 2.7 m/s)	24 h (S)	Low
14	17	10.3	ADO1 (8/6)	A	2.5	Mild aortic turbulence (VMAX 2 m/s)	24 h (S)	Low
15	17	12	CC (8)	A	3.0	Mild aortic turbulence (VMAX 2.3 m/s)	24 h (S)	Low
16	3	3.95	ADO1 (10/8)	A	4.8	Mild aortic turbulence (VMAX 2.9 m/s)	1 y (M)	Low
17	53	17.5	CC (3)	E	0.5	Moderate aortic turbulence (VMAX 3 m/s, no diastolic tail)	24 h (S)	Moderate
18	17	7.9	ADO1 (6/4)	C	2.1	Mild LPA turbulence (VMAX 2.2 m/s)	24 h (S)	Mild

ADO1, Amplatzer ductal occluder 1; CC, Cook Coil; L, late complication; LPA, left pulmonary artery; M, medium-term complication; S, short-term complication; VMAX, maximum velocity.

### Risk factors for complications

Statistical analysis was performed to identify risk factors associated with late aortic obstruction and LPA turbulence compared with the group without complications.

Younger age and increased maximum diameter of the device were associated with late aortic obstruction (see Table 3).

**Table 3:** Risk factors for all complications and late aortic obstruction

Risk factor	Complication free (n = 134)	Late Ao/LPA obstruction (n = 3)	*P*
Weight (kg), mean ± SD	16 ± 8.2	8.3 ± 3.8	0.59
Age (mo), mean ± SD	44.4 ± 31.3	12.6 ± 9.5	**0.013**
Sex (M/F)	47/87	0/3	0.552
Krichenko grading (A, B, C, D, E)	67 A, 3 B, 8 C, 1 D, 52 E	2A/1C	0.257
Type of device used (Cook Coil (CC)/ADO1 (A1)/ADO2 (A2)	79 CC, 51 A1, 4 A2	3 A1	0.165
Maximum diameter of device (mm), mean ± SD	7.7 ± 3.8	16 ± 0	**<0.001**

Bolded *P* values indicates statistical significance (*p* < 0.05).

ADO1, Amplatzer ductal occluder 1; ADO2, Amplatzer ductal occluder 2; Ao, aorta; LPA, left pulmonary artery; SD, standard deviation.

## Discussion

### Early adverse events

The complication rate from our centre is similar to previously reported figures from other studies.^6^^,^^7^ Regarding complications, 83% (15 of 18) were evident either during or immediately after the procedure. Of 156 patients, 5.1% suffered high-grade complications, 1.8% had a residual significant shunt, 1.8% had embolism to the LPA, and 1.2% were unsuccessful and required surgical ligation. These results are comparable to the 5.1% adverse events, 1% residual shunt, 1.2% embolization, and 0.1% unplanned cardiac surgery seen in the IMPACT registry of 1375 patients undergoing ductal occlusion.^8^

Of the 3 patients with devices that embolized to the LPA, all 3 were small CC (two 3-mm and one 5-mm CC). All were successfully retrieved transcutaneously and successfully replaced with larger coils or devices. This reflects earlier practice before the advent of smaller devices such as the piccolo.^3^^,^^9^ Two further patients required subsequent surgical PDA ligation due to inability to fit an appropriate device. These attempts were abandoned and proceeded to surgical ligation, highlighting the need in more difficult cases to have a surgical bailout option. Suggested risk factors associated with occlusion failure include lower body weight, larger ducts, and type C ducts.^10^ Reported failure rates of occlusion devices range from 0% to 1%.^7^^,^^8^^,^^11^

Three patients on follow-up had significant residual flow despite a CC having been fitted necessitating further transcutaneous procedures. It is very common to see insignificant residual flow after occlusion with the observation that, over time, the coil or device endothelializes and clot formation completely impedes ductal flow.^12^ Factors associated with more serious residual leak prompting repeat intervention include larger ductal diameter, the need for multiple coils, and larger preprocedural QP:QS.^13^ Reported repeat procedures due to residual leak range from 1% to 2.5%.^1^^,^^4^

Mild obstruction in the LPA and aorta was seen in the immediate period in 6 patients (3 in the LPA and 3 in the aorta). The obstruction is mild (VMAX <3 m/s), and these patients are being managed expectantly as previously described.^5^^,^^14^^,^^15^ Previous studies have reported that, with growth, clinically silent aortic turbulence resolves;^10^^,^^13^ however, there is a possibility that it may worsen.^16^

### Longer-term adverse events

Interestingly, there was a group of patients, all of whom had 10 × 8 mm ADO1 devices inserted, who developed mid- to late-term obstruction. Of 8 total patients having 10 × 8 mm ADO1 devices inserted, 3 had evidence of medium/late obstruction of the aorta, diagnosed at 1, 3, and 6 years after the procedure, respectively. Increasing device size (measured as the maximum diameter of the retention skirt) was found to be statistically significant in this group (*P* < 0.001); however, the use of the ADO1 itself was not (*P* = 0.165). Flow through the nitinol aortic disc reduces over weeks/months as the disc becomes solid with clot formation ± endothelialization, thereby causing a fixed obstruction of varying severity.^17^

Varying factors could contribute to obstruction being diagnosed later than expected. These were all larger ducts treated with ADO1 devices. The asymmetric design of the ADO1 with a larger aortic retention disc could gradually impede aortic flow.^18^ This contrasts with the symmetrical smaller design of the ADO2.^19^

Patient weight and size are also a potentially important factor; one of the patients with late obstruction was small at 4 kg, although weight was not statistically significant in our cohort. Smaller patients are more likely to have adverse events, especially when under 6 kg and using larger devices or coils.^16^^,^^20^^,^^21^ Failure to demonstrate lower body weight significance in this cohort may be due to the study being underpowered.

Late complications due to transcutaneous device closure are rarely reported in the literature and primarily in the form of case reports. Late embolization has been noted many years after ductal occlusion in the setting of loss to follow-up; it is uncertain whether this was a late complication of unrecognized closer to the time of the procedure.^22^ Haemolysis and occlusion of the femoral artery have also been described as late complications.^23^ Another case of LPA turbulence caused by a 10 × 8 mm ADO1 with a velocity of 2.4 m/s was seen at 2 months after the procedure, worsening at 7 months to 2.7 m/s necessitating stent angioplasty.^24^ Lastly, a case of coarctation of the aorta was caused by a 10 × 8 mm device in a child with trisomy 21 and pulmonary hypertension, diagnosed 3 years after the original procedure.^25^

The complication rate encountered in this study may have been reduced with the introduction of newer devices such as the Amplatzer Vascular Plug or Piccolo devices. Since the period of this study, more devices have become available and more has been learned about device choice. Coils are considered appropriate choices in smaller ducts but are more likely to embolize in larger ducts when compared with devices.^26^ A large single-centre retrospective review found a lower complication rate in children treated primarily with Piccolo devices (80%) reserving the Amplatzer Vascular Plug II/ADOII for larger defects and abandoning coils altogether.^9^ However, in the absence of prospective randomized control trials, comparing devices remains difficult.^27^ Meta-analysis has demonstrated that despite patient weight decreasing, overall procedural success has increased.^28^ The implication of our study is that it may be appropriate to continue follow-up in patients who have undergone device closure with ADO1 devices even in the absence of immediate complications.

### Limitations

This study is limited by being retrospective; clinical and procedural practice has changed over the 10 years during which the study was conducted. A small number of patients were lost to follow-up including one of the late complications at 3 years. Due to the change in imaging software, some of the earlier catheter studies and associated data including precise duct measurements were not available for analysis. Further multicentre collaborative studies with larger numbers and long-term follow-up of such children are needed. Some complications such as minor vascular injury may have been overlooked.

## Conclusions

Most complications after transcutaneous PDA closure occur in the immediate operative or postoperative period. Most patients with an unremarkable procedure without evidence of adverse events at follow-up can safely be discharged at 2 years after the procedure. However, children who have undergone closure at a younger age with a larger diameter device (particularly ADO1) may warrant long-term follow-up due to the potential concern of late aortic turbulence or LPA obstruction.
